# Comparison of Complete Blood Count Results Between K3-EDTA- and MgSO_4_-Anticoagulated Samples Using a DxH800 Analyzer

**DOI:** 10.3390/jcm15124607

**Published:** 2026-06-13

**Authors:** Aurélie Védrenne, Florence Habarou, Tiffany Pascreau, Marc Vasse

**Affiliations:** 1 Biology Department, Foch Hospital, 92150 Suresnes, France; a.vedrenne@hopital-foch.com (A.V.); f.habarou@hopital-foch.com (F.H.); t.pascreau@hopital-foch.com (T.P.); 2 Hémostase Inflammation Thrombose U1176, INSERM, Université Paris-Saclay, 94270 Le Kremlin-Bicêtre, France

**Keywords:** K_3_-ethylene diamine tetracetic acid, magnesium sulphate, complete blood count, differential, DxH800, impedance

## Abstract

**Background/Objectives**: In case of EDTA-induced pseudothrombocytopenia (PTCP), MgSO_4_-anticoagulated tubes are recommended for platelet counting, requiring the collection of an additional tube. The aim of this study was to analyze whether complete blood count (CBC) and differential performed on MgSO_4_-anticoagulated tubes were comparable to the results obtained on K_3_-EDTA samples, and to characterize the stability of the CBC over a 24 h period. **Methods**: In 355 patients (70 with a confirmed PTCP and 285 without PTCP), we compared CBC results obtained on K_3_-EDTA- and MgSO_4_-anticoagulated tubes, using DxH800 analyzers. In 33 cases, a differential was available for both anticoagulants, and for 10 patients, samples were re-analyzed 6, 12, and 24 h after the first determination. **Results**: In the presence or absence of clumps, white blood cell (WBC) count, hematocrit, and mean corpuscular volume (MCV) were slightly lower in MgSO_4_ than in K_3_-EDTA tubes, whereas mean corpuscular hemoglobin concentration (MCHC) was slightly higher. Mean platelet volume (MPV) was significantly lower on MgSO_4_- than on K_3_-EDTA-anticoagulated tubes. Values were highly correlated between both anticoagulants, and mean relative biases (MRBs) were below Ricos’s recommendations, except for MCHC and MPV. For differential, neutrophils were significantly lower on MgSO_4_- in comparison to K_3_-EDTA-anticoagulated tubes (MRB = −2.9%, below Ricos’s optimal bias). The morphology of white blood cells (WBCs) was similar on both anticoagulants. During storage at room temperature, MCV and red cell distribution width increased slightly, but the increase was more pronounced in K_3_-EDTA than in MgSO_4_ tubes. **Conclusions**: CBC and differentials obtained with the DxH 800 analyzer on MgSO_4_-anticoagulated samples are similar to those obtained with K_3_-EDTA, except for MPV.

## 1. Introduction

According to the recommendations of the International Council for Standardization in Hematology (ICSH), ethylenediaminetetraacetic acid (EDTA) is widely used as the preferred anticoagulant for complete blood count (CBC) [1]. EDTA-dependent pseudothrombocytopenia (PTCP) is an in vitro phenomenon of platelet (PLT) clumping that leads to low PLT counts on hematology analyzers. The mechanism is an immunologically mediated phenomenon due to the presence of EDTA-dependent antiplatelet auto-antibodies [2]. Once PCTP has been documented, the main problem is to provide an accurate PLT count, particularly in thrombocytopenic patients, in whom prophylactic PLT transfusions may be necessary. Different strategies have been developed in order to avoid EDTA–PCTP [3]. The anti-aggregating effect of magnesium salts has been described for many years [4,5], and ready-to-use tubes containing magnesium sulfate, Thromboexact™ (Sarstedt, Nürmbrecht, Germany), are commercially available. Recently, a multicenter study by the French-Speaking Cellular Hematology Group (GFHC) indicated that tubes containing citrate, theophylline, adenosine, dipyridamole, or MgSO_4_ are preferred when reevaluating PLT counts in cases of EDTA–PTCP [6]. Use of MgSO_4_-anticoagulated samples, validated only for PLT count, requires collecting an additional volume of blood for the white and red blood cell count, which can be harmful to pregnant women, neonates, or intensive care patients who are often already sampled, leading to increased blood depletion [7]. Recently, it was reported that CBC and differential results obtained on MgSO_4_-anticoagulated samples on Sysmex XN analyzers were comparable to those on K_2_-EDTA samples, except for mean corpuscular volume (MCV), hematocrit, and mean corpuscular hemoglobin concentration (MCHC) [8]. The CBC measurement principles of the Sysmex and Beckman Coulter analyzers are slightly different. Briefly, on Sysmex analyzers, red blood cells (RBCs) are quantified by impedance measurement with hydrodynamic focusing, and erythrocyte indices, including mean corpuscular volume (MCV), are calculated, whereas on Beckman Coulter analyzers, RBCs are quantified by impedance, according to a principle that allows the direct determination of MCV, and other erythrocyte indices are calculated. With the Sysmex technology, leukocytes will be permeabilized in order to allow the labeling of their nucleic acids with polymethine, followed by fluorescence analysis, whereas on Beckman Coulter analyzers, white blood cells (WBCs) are identified by their volume measured by direct impedance, their conductivity, analyzed by radio frequency opacity, and the scatter of a laser beam, as described below [9]. In addition, the use of K_2_- or K_3_-EDTA as an anticoagulant can modify the CBC results [10]. Therefore, the main goal of this study was to assess whether CBC and differential performed on MgSO_4_-anticoagulated tubes were comparable to the results obtained on K_3_-EDTA samples, using the DxH800 analyzer from Beckman Coulter, and to compare the stability of the CBC over a period of 24 h on these anticoagulants.

## 2. Materials and Methods

### 2.1. Samples

From 2 January 2024 to 30 June 2025, EDTA (S-Monovette, K_3_-EDTA, 2.7 mL, Sarstedt), Marnay, France) and Thrombexact™ tubes (Sarstedt) were simultaneously collected from 355 adult patients. All consecutive samples were included, without exclusion criteria. EDTA–PTCP was present in 70 cases, characterized by the presence of an “R” flag on the PLT count and the confirmation of clump presence on a blood smear analyzed at low magnifications (10× and 50×) using a light microscope. For the remaining 285 samples, blood was collected on Thrombexact™ because clinicians suspected EDTA–PTCP based on a previous positive result, but there was no flag on the PLT count, and PLT graphs were normal. CBCs were performed on DxH800 analyzers (Beckman Coulter, Villepinte, France) within 3 h after blood collection. The time elapsed between blood collection and blood analysis was less than 60 min. The time elapsed between the analysis of K_3_-EDTA and MgSO_4_ samples on DxH800 was less than 10 min.

In 33 cases, a differential was also available on both K_3_-EDTA and MgSO_4_ tubes. Lastly, for 10 patients collected from 10 June to 30 June 2025, samples were re-analyzed at 6, 12, and 24 h after the first determination in order to study the stability of the results over time. For this prospective part of the study, all consecutive samples received between Monday and Friday (working days) were included. The tubes were shaken by inversion 5 times and by manual agitation before being placed in the analyzers.

The analyzers were calibrated and maintained according to the manufacturer’s instructions. Internal quality controls (QC) were performed every day, and samples were assayed only if all QC criteria were fulfilled.

Blood smears were performed on a DxH Slidemaker Stainer II (Beckman Coulter), stained with MCDh (Micro Chromatic Detection for Hematology) kit (RAL Diagnostics, Martillac, France), and analyzed on a CellaVision DM 1200 (Lund, Sweden).

### 2.2. Analyzer Characteristics

On DxH 800, RBC are quantified by impedance, and hematocrit is a calculated value derived from the RBC count and from the mean corpuscular volume. Differential is based on “VCS technology”. Briefly, WBC are characterized and identified by their volume “V” (measured by direct impedance), their conductivity “C” (analyzed by conductivity in radio frequency current), and their scatter “S” at different angles of a laser beam [median-angle light scatter (MALS), lower-median-angle light scatter (LMALS), low-angle light scatter (LALS), and upper-median-angle light scatter (UMALS)]. Axial light loss (AL2) measurement analyses cellular transparency. For each leukocyte subpopulation, the mean (MN) and standard deviation (SD) of the mean are calculated, and these 14 parameters (called “cellular positional data” = CPD) allow the identification of WBCs [10].

### 2.3. Statistical Analysis

Data are presented as medians and interquartiles [25th–75th percentiles]. The Shapiro–Wilk test was used to analyze normality and the distribution of the results. Comparisons between counts obtained with K_3_-EDTA and MgSO_4_ anticoagulants were performed with Student’s or Wilcoxon’s paired tests, according to the Gaussian distribution of the parameter tested. To assess stability over time, samples were analyzed at 6, 12, and 24 h after the first measurement (T0). For each value obtained during the kinetic run, the value obtained at T0 was subtracted. The evolution of the variations over time has been evaluated using ANOVA or the Kruskal–Wallis test, depending on the distribution of the values. Multiple comparisons were followed by a Bonferroni post hoc test. The significance level was *p* < 0.05. For method comparison, the Spearman rank-order correlation was used, and the Bland–Altman method was used for the determination of the MRB between K_3_-EDTA- and MgSO_4_-anticoagulated samples. Passing Bablock regressions were also reported. Statistical analyses were performed with Medcalc software, version 19.2 (Ostend, Belgium).

## 3. Results

### 3.1. Comparisons of CBC on EDTA and MgSO_4_-Anticoagulated Samples

The comparison of CBC levels in 355 adult patients (217 females and 138 males; median age 61 years; 25th percentile = 36 years; 75th percentile = 72 years) is reported in Table 1. For WBCs, only 347 values were considered because, in eight cases, a flag was present on the WBC count for K_3_-EDTA samples but absent on MgSO_4_ tubes. This may reflect PLT clumps that can affect the WBC count by impedance, suggesting WBCs of unusually low volumes. The WBC count, hematocrit, and MCV were significantly lower in MgSO_4_ than in K_3_-EDTA tubes, whereas MCHC was higher. For PLTs, we considered only samples without PTCP. The median PLT count was slightly but significantly higher on MgSO_4_ tubes, whereas mean platelet volume (MPV) was lower (Table 1).

Spearman’s coefficients of rank correlations and Passing Bablock regressions (intercepts and slopes) were reported in Table 2 for the entire population (355 samples). Except for MCHC and MPV, the different coefficients of correlation were >0.980. When we analyzed the results based on the presence or absence of PLT clumps, similar results were observed (Table S1). Rank correlations were similar regardless of the presence of clumps.

**Figure 1:**
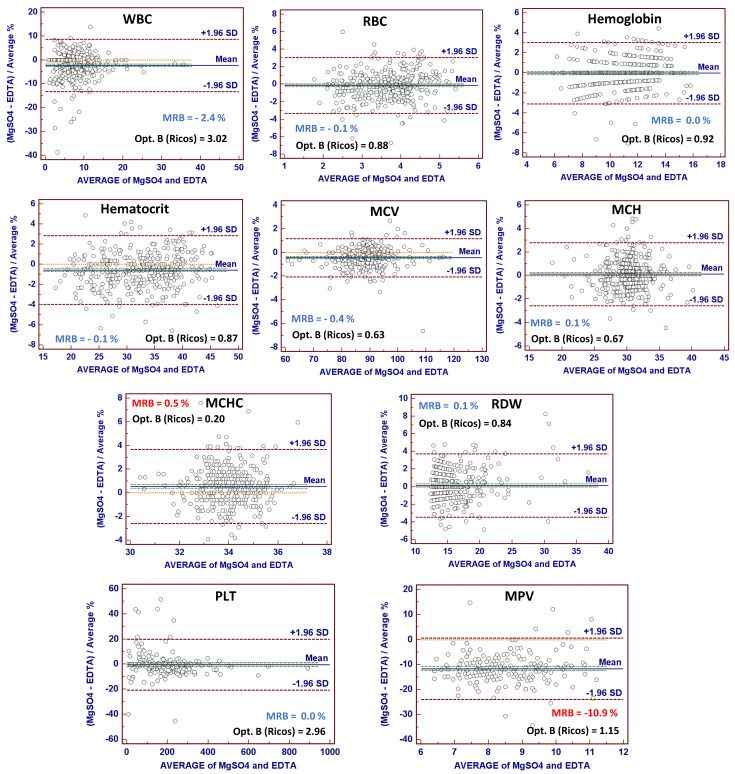
Bland–Altman representation of the different parameters of complete blood count from 355 samples collected either on K_3_-EDTA- (reference) or MgSO_4_-anticoagulated tubes. The mean relative bias (MRB), in %, is indicated for each parameter, as well as the optimal MRB according to Rico’s [11]. They are red when above the optimal MRB. Hatched lines represent the limits of agreement. MCH: mean corpuscular hemoglobin; MCHC: mean corpuscular concentration of hemoglobin; MCV: mean corpuscular volume; MPV: mean platelet volume; Opt. B: optimal bias; PLT: platelet; RBC: red blood cell; RDW: red blood cell distribution width; TBXT: MgSO_4_-anticoagulated tube; WBC: white blood cell.

The mean relative bias (MRB) calculated from Bland–Altman regressions for the different parameters for the whole population is shown in Figure 1. All of them, except for WBCs, MCHC, and MPV, were below Ricos’s optimal bias. For WBCs, however, the MRB was below the desirable bias (6.04%). In contrast, for MCHC and MPV, the MRB values were −0.5% and −10.9%, which were below the minimum bias (0.6% and 1.15%, respectively). When we compare the bias in the presence or absence of PLT clumps, MRB was usually lower in the absence of PLT clumps (Table S1). All of them were below the optimal bias, except for WBCs, in the presence of PLT clumps (MRB = 5.8%) and for MCHC, in the presence or absence of clumps (MRB = −0.8%, and −0.5%, respectively). However, for WBCs, the MRB was below the desirable bias (6.04%). The MRB of MCHC in the presence of clumps was above the minimum bias. Clinical consequences are very weak because if we calculate a new threshold for MCHC on MgSO_4_-anticoagulated samples in the presence of PLT clumps, it is 36.1 g/dL, compared to 36 g/dL on K_3_-EDTA-anticoagulated samples. For MPV, the normal range is between 7.5 and 11.2 fL. Using the Passing Bablok regression, the usual values in tubes anticoagulated with MgSO_4_ would be 6.6 to 10.1 fL

### 3.2. Comparisons of Differentials on K_3_-EDTA- and MgSO_4_-Anticoagulated Tubes

For 33 patients, a differential was performed. Results are presented in Table 3. Only neutrophils were significantly lower on MgSO_4_ tubes in comparison to K_3_-EDTA, but the MRB (−2.9%) observed was below the optimal bias indicated by Ricos’s recommendations (4.62%). For the other lineages, MRB values were also below the optimal bias, except for basophils (MRB = −33.3%; optimal bias = 7.7%; minimal bias 23.1%). However, the MRB of basophils was below the desirable total error (38.5%). Coefficients of correlation showed a good correlation between K_3_-EDTA and MgSO_4_ results but were below 0.9 for eosinophils and basophils.

We also analyzed the consequences of MgSO_4_ use on CPD. The raw values of CPD were first analyzed (Table S2), and when the variations were statistically significant, they were transformed into percentages of variation. For the four lineages, a significant decrease in mean volume of approximately 5% was observed in MgSO_4_ tubes (Table 4), whereas conductivity was not significantly affected. The scatter was differently affected according to the different lineages. For the standard deviations (SD), only four were significantly different between K_3_-EDTA and MgSO_4_ tubes: the SD-V and SD-UMALS of neutrophils [5.5% (2.2 to 9.2) of decrease and 2.4% (−1.0 to 6.7) of increase, respectively], the SD-V of monocytes [13.2% (1.4–21.0)] of decrease, and the SD-MALS-EO –4.2% [(−25.9–3.8)] of decrease.

Results are expressed as a percentage of variation relative to K_3_-EDTA samples, with median and 25th–75th percentiles. AL2: Axial light loss; LALS: low-angle light scatter; LMALS: lower-median-angle light scatter; MALS: median-angle light scatter; MN: mean; n.s: not significant; UMALS: upper-median-angle light scatter; V: volume.

**Figure 2:**
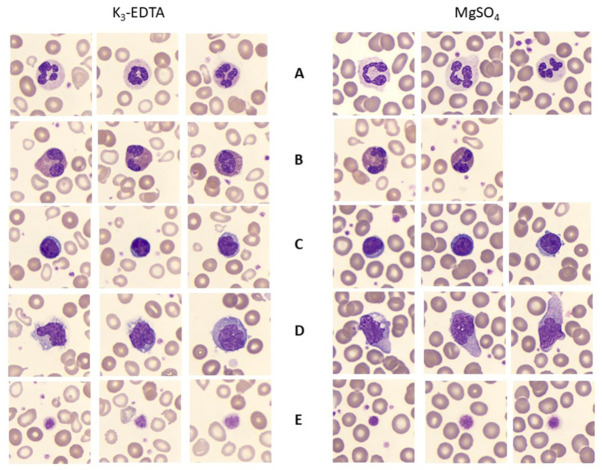
Comparison of the morphology of leukocytes when the smear was performed from K_3_-EDTA- or MgSO_4_-anticoagulated tubes. Blood smears were performed on a DxH Slidemaker Stainer II, stained with an MCDh (Micro Chromatic Detection for Hematology) kit and analyzed on a CellaVision DM 1200. (**A**) Neutrophils; (**B**) eosinophils; (**C**) lymphocytes; (**D**) monocytes; (**E**) platelets.

Despite subtle variations detected by the analyzers, the morphology and staining of the different cell types were similar between K_3_-EDTA- and MgSO_4_-anticoagulated tubes when smears were stained with MCDh (Figure 2).

### 3.3. Long-Term Stability of Samples Collected on MgSO_4_-Anticoagulated Tubes

Long-term stability of CBC was evaluated over a 24 h period. Ten samples were analyzed at times 6, 12, and 24 h after the first analysis (T0). All samples were maintained at room temperature (21 to 25 degrees Celsius) throughout the evaluation period. All parameter results for MgSO_4_ tubes were compared to the K_3_-EDTA results obtained at the same time intervals. For PLT count and MPV, only eight values were considered due to EDTA–PTCP in two patients. At T0, as previously detailed, on this small series, WBCs were significantly higher in K_3_-EDTA samples than in MgSO_4_ [10.1 (7.5–13.7) versus 9.9 × 10^9^/L (7.4–12.9), respectively, *p* = 0.0448], MCV [86 (84.4–90.4) versus 85.5 fL (84.1–90.2), respectively, *p* = 0.0448] as well as MPV [9.0 (8.5–9.5) versus 8.2 fL (7.2–8.6), *p* < 0.0001] whereas other parameters of CBCs were not significantly different.

**Figure 3:**
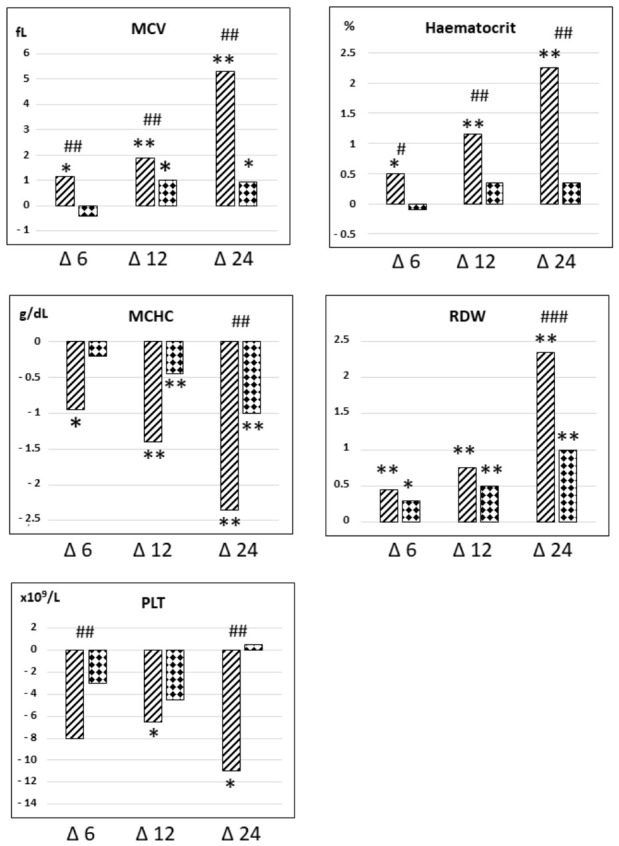
Variations in complete blood count parameters when samples are collected either in K_3_-EDTA tubes (oblique lines) or in MgSO_4_ tubes (diamonds) and re-analyzed 6 (Δ 6), 12 (Δ 12), or 24 (Δ 24) hours after the first analysis (T0). *: comparison to T0; ^#^ comparison between K3-EDTA and MgSO_4_ samples. *: *p*< 0.05; ** *p* <0.01; ^##^: *p* < 0.01; ^###^: *p* < 0.001.

WBCs, RBCs, hemoglobin, and MCH levels were not significantly affected by storage at room temperature during a 24 h period, either on K_3_-EDTA- or MgSO_4_-anticoagulated tubes. In contrast, MCV, hematocrit, RDW, and MCMH began to increase significantly from the 6th hour of storage on K_3_-EDTA-anticoagulated samples and continued to increase over time, whereas MCHC decreased (Figure 3). On MgSO_4_-anticoagulated samples, RDW also increased slightly from the 6th hour, but after 24 h, the increase was significantly higher (*p* = 0.004) on K_3_-EDTA tubes compared to MgSO_4_-anticoagulated samples. MCV slightly increased from the 12th hour on MgSO_4_-anticoagulated tubes, but the increase was less marked than on K_3_-EDTA, and this small increase in MCV did not significantly affect hematocrit as observed for K_3_-EDTA. MCHC also decreased significantly from the 12th hour of storage on MgSO_4_-anticoagulated tubes, but the decrease was significantly smaller than that observed in K_3_-EDTA samples. Concerning PLTs, their levels remained stable when blood was collected in MgSO_4_ tubes, whereas they decreased over time in K_3_-EDTA-anticoagulated tubes. MPV was not significantly affected regardless of the anticoagulant used.

## 4. Discussion

PTCP induced by EDTA is a well-known artifact that can lead to an erroneous result of the PLT count, and the falsely low PLT count may trigger unnecessary, expensive, and even invasive diagnostic or therapeutic procedures. MgSO_4_, previously used for PLT counting in capillary blood, has been shown to be an efficient anticoagulant to avoid PLT clump formation in case of EDTA-induced PTCP [12,13,14] and has been proposed as the best alternative for PLT quantification in patients with EDTA-induced PTCP [6]. Unfortunately, the use of this specific anticoagulant requires the collection of another EDTA-anticoagulated sample for CBC parameters (WBC, RBC, hemoglobin, and erythrocyte indices). Therefore, the aim of the study was to determine whether CBC obtained on commercially available MgSO_4_-anticoagulated tubes could be relevant.

On a retrospective series of 355 samples, for which K_3_-EDTA and MgSO_4_ tubes were collected simultaneously, and analyzed at similar times on analyzers based on impedance technology (DxH 800), RBC counts, hemoglobin levels, MCH, and RDW were not significantly different, and of course, significantly correlated. In contrast, MCV and hematocrit were significantly lower on MgSO_4_-anticoagulated samples, as well as MPV. These variations in MCV and hematocrit are very modest and do not affect the interpretation of a possible anemia. In contrast, the variations in MPV are highly significant for clinical interpretation and clearly warrant the establishment of new reference values. A swelling of PLTs when tubes are collected on EDTA has been previously described [15,16] and is less pronounced in MgSO_4_-anticoagulated blood [17,18], because magnesium has been shown to inhibit platelet activation by different mechanisms [19]. As in our study, swelling of RBC was also observed in EDTA samples kept at room temperature over time [18,19]. In contrast, the swelling of RBCs when MgSO_4_ was used as an anticoagulant was weak and significant only from the 12th hour after the first analysis and remained stable (1 fL after a 24 h storage at room temperature for MgSO_4_- versus 5.3 fL for K_3_-EDTA-anticoagulated samples). The difference between MgSO_4_ and K_3_-EDTA is possibly due to the fact that magnesium ions were shown to regulate the stability of the RBC membrane [20]. The slight but significant difference in MCV at T0 (89.8 fL for K3-EDTA versus 89.3 fL for MgSO_4_ samples, *p* < 0.0001) could be due to rapid swelling in the K_3_-EDTA tubes between phlebotomy and blood analysis. If it was not recorded in this study, the median time between blood collection and analysis is approximately 60 min in our hospital. In addition to the variations in MCV, hematocrit, and RDW were slightly lower on MgSO_4_ tubes than in K_3_-EDTA, of course, in relation to the swelling of RBCs in K_3_-EDTA-anticoagulated tubes, and the differences between samples collected on K_3_-EDTA and MgSO_4_ become more marked over time, with the lowest variations being observed for the MgSO_4_-anticoagulated tubes. Similar variations in MCV, hematocrit, and MCHC were also reported in a recent study that examined 250 samples and compared K_2_-EDTA and MgSO_4_ samples analyzed on Sysmex XN analyzers [8].

Regarding WBCs, our study showed lower levels in MgSO_4_ samples than in K_3_-EDTA samples. A similar result was previously observed by Schuff-Werner et al. [12] in 21 patients: the mean WBC count was 8.25 × 10^9^/L on EDTA versus 7.99 × 10^9^/L on MgSO_4_ samples. As in our study, neutrophils were also significantly lower (mean level of WBC was 5.61 × 10^9^/L on EDTA versus 5.45 × 10^9^/L on MgSO_4_ samples, *p* = 0.0012) on MgSO_4_ samples than in the EDTA-anticoagulated tube, whereas lymphocyte and monocyte counts were not significantly different. The EDTA salt used was not reported (K_2_ or K_3_)_,_ and most of the analyzers were from Beckman Coulter. At least three hypotheses can be proposed. Since neutrophils have a significantly smaller volume in MgSO_4_ samples compared to EDTA tubes, it can be inferred that some neutrophils in MgSO_4_ samples have a small volume and are excluded from the differential algorithm. However, this does not explain that the total WBC count is also lower in MgSO_4_ samples, since lymphocytes are smaller than neutrophils; therefore, small neutrophils should be counted in the total WBC count. A second hypothesis would be that neutrophils aggregate in the presence of MgSO_4_, but no flag was observed, suggesting an erroneous leukocyte count. Lastly, it can be observed that some neutrophils are lysed in the presence of MgSO_4_. In contrast, Soulard et al. [8] did not observe significant bias when WBC count and differential were performed on K2-EDTA tubes analyzed on the Sysmex XN analyzer. At this time, it is not clear whether these different results are due to the use of different potassium salts or to differences in measurement methods between the two analyzers.

Interestingly, on blood smears, the morphology of the blood cells collected in MgSO_4_ tubes was comparable to that of samples collected in K_3_-EDTA, consistent with the results of Schuff-Werner et al. [12]. In contrast, in 15 patients, Choccalingam et al. concluded that the quality of the Leishman-stained smears collected in MgSO_4_-anticoagulated tubes was below average when compared with EDTA smears for WBCs and RBCs, while PLT morphology was comparable to that of EDTA smears [21]. This discrepancy can be due to the use of a “home-made” anticoagulant, derived from a commercially available MgSO_4_ solution used for patient injection (Magneon; Neon Laboratories Ltd., Mumbai, India), while Schuff-Werner et al. and we used a commercially available MgSO_4_-anticoagulated tube, Thrombexact™. Interestingly, Choccalingam et al. also described similar ranges of WBCs and RBCs between EDTA and MgSO_4_ tubes, but a higher MCV on MgSO_4_-anticoagulated tubes. Unfortunately, the final concentration of MgSO_4_ was not reported in their study, making comparisons between their results and ours difficult. They also concluded that further studies on different concentrations of MgSO_4_ are required in order to use MgSO_4_ as an anticoagulant for CBCs. Indeed, it was previously observed that MCV variations were dependent on the concentrations of K_2_-EDTA used, with higher concentrations of K_2_-EDTA (16 g/L) associated with higher MCV than those observed with low concentrations (2 g/L) of K_2_-EDTA [22].

Importantly, there was a high correlation between the different CBC parameters obtained with MgSO_4_- or EDTA-anticoagulated tubes, and CBC values remained stable during a 24 h period, with better stability on MgSO_4_-anticoagulated tubes, since cell swelling was reduced.

The strength of our study, in real life, is that it included a large number of samples, with wide extreme values of WBCs and RBCs (from 0.8 × 10^9^/L to 99.8 × 10^9^/L for WBC, from 1.76 × 10^9^/L to 5.42 × 10^9^/L for RBC, from 61.0 fL to 116.2 fL for MCV). This work also has several limitations: It is a monocentric, retrospective study, at least concerning the comparison of CBC parameters. In addition, for this study, we compared samples from patients with suspicion of PTCP, mainly because patients had prior PTCP. The PTCP was not confirmed in 285 cases and was considered “normal”. This could introduce a selection bias and reduce the generalizability of the findings to the wider patient population. This study was performed on a DxH 800 analyzer, based on the impedance method. Unfortunately, comparisons of hematology analyzers from different manufacturers have shown an overall moderate agreement between the analyzers [23]. Therefore, we cannot exclude that the good agreement observed for RBC count or hemoglobin levels on a DxH 800 analyzer is also observed for other analyzers that enumerate RBCs optically, for example. Lastly, for differential, we observed good global agreement between the two anticoagulants for “normal” cells, but we did not test patients with activated lymphocytes or immature granular leukocytes. In addition, the number of samples tested for differential, although sufficient for a method validation [24], is low, particularly for eosinophils and basophils, since they were undetectable in some samples. Therefore, this preliminary study requires additional studies to confirm these data, but they are nevertheless in agreement with the recent study using samples collected on K2-EDTA and analyzed on XN analyzers [8]. Such a limitation applies, of course, regarding the stability study.

## 5. Conclusions

In conclusion, we suggest that CBC and differentials obtained from the DxH 800 analyzer on MgSO_4_-anticoagulated samples are similar to those obtained using K_3_-EDTA anticoagulated tubes, both for samples with or without PLT clumps. As previously observed for PLTs [8,13,25], the use of MgSO_4_ as an anticoagulant does not significantly increase RBC volume during storage when using impedance technology, and it could be employed in situations where an accurate measurement of the MCV is necessary, for example, for the detection of α-thalassemia-1 and β-thalassemia traits in pregnant women [26]. Lastly, the MRB values observed for some parameters were only marginal, within the range of test variability and below the desirable or optimal Ricos’s specifications [11]. The use of MgSO_4_ on DxH800 produces results comparable to those obtained with K_3_-EDTA, except for MPV, for which it is necessary to establish new reference values.

## Figures and Tables

**Table 1:** Comparison of complete blood count parameters of 355 patients when blood was collected both on K_3_-EDTA- and on MgSO_4_-anticoagulated tubes.

	K_3_-EDTA	MgSO_4_	*p*
White Blood Cells (×10^9^/L) *	**7.4** [5.2–10.0]	**7.1** [5.1–9.7]	<0.0001
Red Blood Cells (×10^12^/L)	**3.75** [3.2–4.2]	**3.75** [3.15–4.17]	0.2806
Hemoglobin (g/dL)	**11.3** [9.4–12.8]	**11.3** [9.4–12.8]	0.8897
Hematocrit (%)	**33.3** [27.7–37.5]	**33.0** [27.4–37.4]	<0.0001
Mean Corpuscular Volume (fL)	**89.8** [85.0–92.8]	**89.3** [84.5–92.5]	<0.0001
Mean Corpuscular Hemoglobin (pg)	**30.4** [28.7–31.7]	**30.4** [28.8–31.8]	0.3165
Mean Corpuscular Hemoglobin Concentration (g/dL)	**33.9** [33.2–34.6]	**34.1** [33.5–34.8]	<0.0001
Red Cell Distribution Width	**14.9** [13.7–17.5]	**14.8** [13.7–17.4]	0.1669
Platelets (×10^9^/L) **	**150** [80–259]	**151** [83–248]	0.0001
Mean Platelet Volume (fL) **	**8.9** [8.2–10]	**8.0** [7.3–8.9]	<0.0001

* only for the 347 samples without flag on WBC when counted on K_3_-EDTA tubes; ** only for the 285 samples without platelet clumps. Results are expressed as median (in bold) and [25th–75th percentiles].

**Table 2:** Spearman’s coefficients of rank correlations and Passing Bablok regressions of 355 samples anticoagulated with K_3_-EDTA or MgSO_4_.

	Spearman’s Coefficients of Rank Correlations [95% CI]	Slope [95% CI]	Intercept [95% CI]
White Blood Cells (×10^9^/L) *	0.993 [0.991–0.994]	0.9872 [0.9767–1.0000]	−0.0282 [−0.1000–0.0395]
Red Blood Cells (×10^12^/L)	0.996 [0.996–0.997]	1.0109 [1.0000–1.0192]	−0.0420 [−0.0714–0.0000]
Hemoglobin (g/dL)	0.996 [0.949–0.969]	1.0000 [1.0000–1.0161]	0.0000 [−0.1758–0.0000]
Hematocrit (%)	0.995 [0.994–0.996]	1.0000 [1.0000–1.0153]	−0.2000 [−0.6557–−0.2000]
Mean Corpuscular Volume (fL)	0.992 [0.990–0.993]	1.0081 [1.0000–1.0192]	−1.1008 [−2.0611–0.4000]
Mean Corpuscular Hemoglobin (pg)	0.980 [0.976–0.984]	1.0000 [0.9429–1.0133]	0.0000 [−0.4040–0.0000]
Mean Corpuscular Hemoglobin Concentration (g/L)	0.841 [0.808–0.869]	1.0000 [0.9429–1.0357]	0.2000 [−1.0375–2.1229]
Red Cell Distribution Width	0.991 [0.988–0.992]	1.0000 [1.0000–1.0156]	0.0000 [−0.2273–0.0000]
Platelets (×10^9^/L) **	0.996 [0.995–0.997]	0.9697 [0.9605–0.9791]	2.2422 [1.2487–3.5066]
Mean Platelet Volume (fL) **	0.913 [0.891–0.930]	0.9375 [0.8889–1.0000]	−0.3937 [−1.0000–0.0167]

* only for the 347 samples without flag on WBC when counted on K3-EDTA tubes; ** only for the 285 samples without platelet clumps.

**Table 3:** Comparison of differentials of 33 patients when blood was collected both on K_3_-EDTA- and on MgSO_4_-anticoagulated tubes.

Cells (×10^9^/L)	K_3_-EDTA	MgSO_4_	*p*	Spearman’s Coefficients of Rank Correlations [95% CI]	Mean Relative Bias (Bland–Altman) (%)	Slope [95% CI]	Intercept [95% CI]
WBC	**8.3** [5.1–9.8]	**8.1** [5.4–9.2]	0.0151	0.977 [0.954–0.989]	−2.9	0.9813 [0.9310–1.0128]	−0.0346 [−0.1449–0.2483]
Neutrophils	**5.4** [3.2–7.4]	**5.4** [3.3–7.3]	0.0038	0.991 [0.982–0.996]	−2.6	0.9706 [0.9250–1.0000]	0.0147 [−0.1000–0.1525]
Eosinophils	**0.1** [0.0–0.1]	**0.1** [0.0–0.1]	0.7285	0.854 [0.726–0.926]	−9.8 *	1.0000 [1.0000–1.0000]	0.0000 [0.0000–0.0000]
Basophils	**0.0** [0.0–0.1]	**0.0** [0.0–0.1]	0.8695	0.899 [0.827–0.942]	−33.3 **	1.0000 [1.0000–1.0000]	0.0000 [0.0000–0.0000]
Lymphocytes	**1.2** [0.8–1.6]	**1.2** [0.8–1.6]	0.8596	0.886 [0.971–0.993]	−0.3	1.0000 [0.9091–1.0000]	0.0000 [0.0000–0.0818]
Monocytes	**0.6** [0.4–0.8]	**0.6** [0.4–0.8]	0.4405	0.945 [0.971–0.993]	−2.6	1.0000 [0.8333–1.0000]	0.0000 [0.0000–0.8333]

* Calculated for 22 samples since in 11 cases, no eosinophils were detected in both K_3_-EDTA- and MgSO_4_-anticoagulated tubes. ** Calculated for 12 samples since in 21 cases, no basophils were detected in both K_3_-EDTA- and MgSO_4_-anticoagulated tubes.

**Table 4:** Percentages of variation in the different cellular population data of leukocytes between K_3_-EDTA- and MgSO_4_-anticoagulated tubes.

Cells (×10^9^/L)	MN-V	MN-MALS	MN-UMALS	MN-LMALS	MN-LALS	MN-AL2
Neutrophils	**−5.2** [−7.1–−4.1]	**3.8** [0.7–4.9]	**1.4** [−0.3–3.1]	**5.9** [1.5–8.3]	**9.1** [5.1–12.6]	**−3.8** [−9.0–−2.4]
Eosinophils	**−5.1** [−7.6–−2.2]	**2.3** [1.9–3.5]	**1.8** [1.0–3.3]	**3.2** [2.1–4.2]	n.s	**−4.0** [−9.7–−0.8]
Lymphocytes	**−2.4** [−3.7–−1.2]	**−2.9** [−6.0–0.0]	**−6.3** [−10.5–−3.5]	n.s	**5.7** [2.1–7.9]	n.s
Monocytes	**−6.7** [−8.2–−3.0]	n.s	**−5.1** [−7.3–−2.2]	**8.8** [5.2–9.9]	**21.0** [13.2–28.5]	n.s

## Data Availability

The data that support the findings of this study are available from the corresponding author (M.V.) upon reasonable request.

## Supplementary Materials

The following supporting information can be downloaded at: https://www.mdpi.com/article/10.3390/jcm15124607/s1, **Table S1**: Comparison of complete blood count of samples anticoagulated either with K_3_-EDTA or MgSO_4_ according to the presence (n = 70) or the absence of platelet clumps (n = 285). Spearman’s coefficients of rank correlations and Passing–Bablock regressions between both anticoagulants; **Table S2**: Comparison of the cellular population data of 33 patients whose blood was collected in K3-EDTA- or MgSO_4_-anticoagulated tubes.

## Abbreviations

The following abbreviations are used in this manuscript:

AL2	Axial light loss
CBC	Complete blood count
CPD	Cellular positional data
EDTA	Ethylene diamine tetracetic acid
GFHC	French-Speaking Cellular Hematology Group
LALS	Low-angle light scatter
LMAL	Lower-median-angle light scatter
M	Mean
MALS	Median-angle light scatter
MCHC	Mean corpuscular hemoglobin concentration
MCV	Mean corpuscular volume
MPV	Mean platelet volume
MRB	Mean relative bias
PLT	Platelet
PTCP	Pseudothrombocytopenia
RBC	Red blood cell
SD	Standard deviation
UMALS	Upper-median-angle light scatter
WBC	White blood cell
