# Supplementary material for: Comparison of Complete Blood Count Results Between K3-EDTA- and MgSO4-Anticoagulated Samples Using a DxH800 Analyzer
Source: J Clin Med. 2026 Jun 13;15(12):4607. doi: 10.3390/jcm15124607 (PMC13302607; doi:10.3390/jcm15124607)
Supplement: Supplementary file 1 [file jcm-15-04607-s001.zip › JCM_S-table S1.pdf]

Table S1: Comparison of complete blood count of samples anticoagulated either with K_3-EDTA or $MgSO_4$ according to the presence (n = 70) or the absence of platelet clumps (n = 285). Spearman's coefficients of rank correlations and Passing-Bablock regressions between both anticoagulants

	Platelet clumps	K_3-EDTA	$MgSO_4$	*p*	Spearman's coefficients of rank correlations [95% CI]	Mean bias % (Bland-Altman)	Opt. bias (%) [desir. bias – min.-bias] (Ricos)	Slope [95% CI]	Intercept [95% CI]
White Blood Cells (x 10^9/L)	Yes	7.2 [4.3 – 9.2]	6.8 [4.1 – 8.6]	< 0.0001	0.977 [0.961 – 0.986]	5.8 [-9.1 – 20.6]	3.02 [6.04 – 9.06]	0.9500 [0.9032 – 0.9855]	0.0300 [- 0.1812 – 0.3097]
	No	7.4 [5.2 – 10.0]	7.2 [5.2 – 9.9]	< 0.0001	0.996 [0.995 – 0.997]	1.6 [-7.6 – 10.9]		0.9910 [0.9787 – 1.0000]	- 0.0356 [- 0.1000 – 0.0489]
Red Blood Cells (x 10^9/L)	Yes	4.0 [3.4 – 4.2]	3.9 [3.4 – 4.1]	0.6738	0.996 [0.993 – 0.997]	0.3 [- 2.5 – 3.1]	0.88 [1.77 – 2.65]	1.0000 [0.9877 – 1.0256]	- 0.0100 [- 0.1000 – 0.0320]
	No	3.7 [3.1 – 4.2]	3.7 [3.1 – 4.2]	0.1430	0.997 [0.996 – 0.997]	0.1 [-3.2 – 3.4]		1.0132 [1.0000 – 1.0223]	- 0.0484 [- 0.0808 – 0.0000]
Haemoglobin (g/dL)	Yes	11.3 [9.8 – 12.7]	11.3 [9.8 – 12.6]	0.6507	0.997 [0.995 – 0.998]	0.0 [- 2.7 – 2.7]	0.92 [1.77 – 2.65]	1.0000 [1.0000 – 1.0222]	0.0000 [- 0.2422 – 0.0000]
	No	11.3 [9.4 – 12.8]	11.3 [9.4 – 12.8]	0.9467	0.997 [0.996 – 0.997]	0.0 [- 3.1 – 3.2]		1.0000 [1.0000 – 1.0192]	0.0000 [- 0.2106 – 0.0000]
Haematocrit (%)	Yes	34.2 [28.4 – 37.5]	33.6 [28.2 – 36.8]	0.0002	0.992 [0.987 – 0.995]	0.8 [-2.1 – 3.7]	0.87 [1.74 – 2.61]	1.0000 [0.9862 – 1.0258]	- 0.2000 [- 1.0510 – 0.2214]
	No	33.0 [27.6 – 37.4]	32.9 [27.2 – 37.4]	< 0.0001	0.996 [0.995 – 0.997]	0.5 [-3.0 – 4.1]		1.0070 [1.0000 – 1.0189]	- 0.3678 [- 0.7509 – 0.2000]

Mean Corpuscular volume (fL)	Yes	89.6 [82.2 – 91.3]	89.0 [81.5 – 91.0]	0.0002	0.986 [0.978 – 0.991]	0.4 [-1.2 – 2.1]	0.63 [1.26 – 1.89]	1.0248 [1.0000 – 1.0500]	- 2.5555 [- 4.7450 – -0.4000]
	No	89.8 [85.8 – 93.2]	89.5 [85.5 – 92.7]	< 0.0001	0.992 [0.990 – 0.994]	0.5 [-1.2 – 2.1]		1.0000 [1.0000 – 1.0167]	- 0.4000 [- 1.8467 – - 0.4000]
Mean Corpuscular Haemoglobin (pg)	Yes	29.7 [27.0 – 30.7]	30.0 [27.3 – 31.0]	0.0373	0.990 [0.984 – 0.994]	- 0.4 [-3.2 – 2.4]	0.67 [1.35 – 2.02]	1.0000 [0.9667 – 1.0278]	0.1000 [- 0.7750 – 1.0967]
	No	30.5 [29.1 – 32.0]	30.6 [29.1 – 31.8]	0.6619	0.981 [0.977 – 0.985]	0.0 [-2.7 – 2.6]		1.0000 [1.0000 – 1.0196]	0.0000 [- 0.6039 – 0.0000]
Mean Corpuscular Haemoglobin Concentration (g/L)	Yes	33.5 [32.9 – 34.3]	33.8 [33.2 – 34.6]	0.0002	0.871 [0.799 – 0.918]	- 0.8 [- 4.0 – 2.4]	0.20 [0.40 – 0.60]	0.9535 [0.8333 – 1.0571]	1.7869 [- 1.6971 – 5.8333]
	No	34.0 [33.3 – 34.7]	34.2 [33.5 – 34.8]	< 0.0001	0.836 [0.798 – 0.868]	- 0.5 [- 3.6 – 2.6]		1.0000 [0.9500 – 1.0625]	0.1000 [- 1.9875 – 1.8500]
Red cell Distribution Width	Yes	15.8 [13.4 – 18.5]	15.9 [13.4 – 19.1]	0.2759	0.988 [0.981 – 0.93]	- 0.4 [- 5.4 – 4.5]	0.84 [167 – 2.51]	1.0408 [1.0000 – 1.0714]	- 0.5592 [- 1.0500 – 0.0000]
	No	14.9 [13.7 – 16.9]	14.8 [13.7 – 17.0]	0.3953	0.989 [0.987 – 0.992]	- 0.0 [- 3.6 – 3.6]		1.0000 [1.0000 – 1.0000]	0.0000 [- 0.0000– 0.0000]

desir.: desirable; min: minimum; Opt: optimal

p: difference between levels obtained on K_3-EDTA or $MgSO_4$-anticoagulated tubes (paired Student or Wilcoxon test, according to the distribution of the levels)
