# Supplementary material for: Comparison of Complete Blood Count Results Between K3-EDTA- and MgSO4-Anticoagulated Samples Using a DxH800 Analyzer
Source: J Clin Med. 2026 Jun 13;15(12):4607. doi: 10.3390/jcm15124607 (PMC13302607; doi:10.3390/jcm15124607)
Supplement: Supplementary file 1 [file jcm-15-04607-s001.zip › JCM_table S2.pdf]

Table S2: Comparison of the cellular population data of 33 patients whose blood was collected in K_3-EDTA or $MgSO_4$-anticoagulated tubes.

**Neutrophils**	**K_3-EDTA**	**$MgSO_4$**	***p***
MN-V-NE	149 [144 – 156]	141 [136 – 147]	< 0.0001
SD-V-NE	17.5 [16.7 – 21.1]	16.7 [16.1 – 23.1]	0.0147
MN-C-NE	145 [143 – 147]	145 [143 – 148]	0.7108
SD-C-NE	4.8 [4.4 – 5.5]	5.1 [4.8 – 5.9]	0.0313
MN-MALS-NE	139 [136 – 143]	141 [136 – 149]	0.0003
SD-MALS-NE	10.3 [9.5 – 11.1]	10.3 [9.9 – 12.3]	0.1952
MN-UMALS-NE	138 [132 – 143]	137 [131 – 145]	0.0239
SD-UMALS-NE	10.8 [10.5 – 11.8]	11.3 [10.7 – 12.9]	0.0267
MN-LMALS-NE	133 [127 – 140]	139 [133 – 147]	0.0002
SD-LMALS-NE	12.9 [11.8 – 14.1]	12.1 [11.5 – 14.2]	0.6877
MN-LALS-NE	159 [150 – 167]	176 [167 – 182]	0.0002
SD-LALS-NE	29.1 [27.4 – 31.6]	29.0 [28.0 – 34.5]	0.5259
MN-AL2-NE	149 [136 – 155]	140 [130 -145]	< 0.0001
SD-AL2-NE	13.9 [11.8 – 15.8]	12.8 [11.0 – 16.2]	0.1807
**Lymphocytes**			
MN-V-LY	90 [85 - 94]	86 [84 - 92]	0.0003
SD-V-LY	15.2 [14.1 – 18.3]	14.6 [13.9 – 16.6]	0.2021
MN-C-LY	113 [112 – 114]	114 [112 – 115]	0.5888
SD-C-LY	7.9 [7.3 – 10.8]	9.7 [7.4 – 11.8]	0.1831

MN-MALS-LY	73<br>[70 – 79]	71<br>[69 – 74]	0.0015
SD-MALS-LY	15.9<br>[15.5 – 17.5]	16.3<br>[15.4 – 19.7]	0.7956
MN-UMALS-LY	73<br>[69 – 77]	67<br>[65 – 71]	< 0.0001
SD-UMALS-LY	19.1<br>[18.3 – 21.5]	19.4<br>[18.2 – 22.0]	0.3538
MN-LMALS-LY	67<br>[62 – 72]	68<br>[64 – 70]	0.8732
SD-LMALS-LY	19.0<br>[17.9 – 19.5]	18.9<br>[17.9 – 22.5]	0.2994
MN-LALS-LY	38<br>[35 – 42]	40<br>[37 – 42]	< 0.0001
SD-LALS-LY	10.8<br>[10.0 -13.3]	10.9<br>[9.9 -13.3]	0.7009
MN-AL2-LY	73<br>[65 – 90]	66<br>[64 – 75]	< 0.0001
SD-AL2-LY	14.9<br>[12.9 – 17.4]	13.3<br>[12.3 – 16.9]	0.3677
**Monocytes**			
MN-V-MO	177<br>[175 – 184]	167<br>[164– 174]	< 0.0001
SD-V-MO	20.5<br>[19.0 –23.0]	18.8<br>[16.2 – 22.0]	0.0006
MN-C-MO	122<br>[120 – 124]	122<br>[121 – 124]	0.1057
SD-C-MO	5.3<br>[4.5 – 6.1]	4.9<br>[4.1 – 6.3]	0.7956
MN-MALS-MO	90<br>[86 – 91]	91<br>[89 – 92]	0.0877
SD-MALS-MO	10.8<br>[10.1 -12.4]	10.9<br>[10.3 -12.8]	0.4832
MN-UMALS-MO	98<br>[96 – 103]	95<br>[91 – 98]	< 0.0001
SD-UMALS-MO	11.2<br>[10.0 – 13.7]	11.3<br>[10.7 – 12.5]	0.7009
MN-LMALS-MO	75<br>[72 – 79]	83<br>[79 – 85]	< 0.0001
SD-LMALS-MO	14.2<br>[13.6 -16.2]	14.1<br>[13.1 -16.6]	0.9929

MN-LALS-MO	80<br>[69 - 84]	103<br>[91 - 110]	< 0.0001
SD-LALS-MO	25.7<br>[24.3 – 28.2]	24.5<br>[22.2 – 29.7]	0.6106
MN-AL2-MO	130<br>[117 -156]	123<br>[118 -134]	0.0806
SD-AL2-MO	19.0<br>[16.7 – 21.5]	17.3<br>[16.4 – 20.6]	0.1098
**Eosinophils**			
MN-V-EO	156<br>[147 -161]	148<br>[142 -155]	< 0.0001
SD-V-EO	16.7<br>[14.8 – 18.8]	14.7<br>[13.7 – 18.0]	0.0879
MN-C-EO	147<br>[145 -149]	148<br>[147 -150]	0.1254
SD-C-EO	4.1<br>[3.9 – 4.9]	4.1<br>[3.5 – 10.9]	0.3214
MN-MALS-EO	198<br>[194 -204]	202<br>[199 -208]	< 0.0001
SD-MALS-EO	8.8<br>[7.9 – 10.8]	9.3<br>[8.4 – 10.5]	0.3127
MN-UMALS-EO	210<br>[204 - 215]	214<br>[208 - 218]	< 0.0001
SD-UMALS-EO	10.0<br>[9.2 – 11.5]	10.8<br>[9.1 – 13.0]	0.0948
MN-LMALS-EO	182<br>[180 -180]	189<br>[185 -193]	< 0.0001
SD-LMALS-EO	10.7<br>[9.8 – 12.3]	10.5<br>[9.4 – 11.8]	0.0342
MN-LALS-EO	154<br>[122 -164]	158<br>[149 -168]	0.5878
SD-LALS-EO	40.4<br>[38.1 – 43.6]	40.6<br>[36.1 – 45.2]	0.4914
MN-AL2-EO	126<br>[122 -131]	120<br>[114 -125]	0.0001
SD-AL2-EO	11.2<br>[10.2 – 13.5]	11.0<br>[9.7 – 13.2]	0.6815
